# Early Postural Instability in Parkinson's Disease: A Biomechanical Analysis of the Pull Test

**DOI:** 10.1155/2019/6304842

**Published:** 2019-10-24

**Authors:** Javier Ricardo Pérez-Sánchez, Francisco Grandas

**Affiliations:** Movement Disorders Unit, Neurology Department, Hospital General Universitario Gregorio Marañón, C./Doctor Esquerdo 46, 28007 Madrid, Spain

## Abstract

Postural instability in Parkinson's disease (PD) is commonly assessed by the pull test. This clinical test may be biased by the variability of the pull force applied. Our objective was to study the postural responses elicited by reproducible pull forces in healthy subjects and PD patients at different stages of the disease. We performed a multimodal approach that included a systematic analysis of the pull force needed to reach the backward limit of stability (FBLoS) assessed by mechanically produced forces, the displacements of the center of pressure (CoP) recorded on a force platform, and the latencies and patterns of activation of the stabilizing muscles. Comparisons between groups were performed by univariate and multivariate statistical analyses. Sixty-four healthy subjects and 32 PD patients, 22 Hoehn–Yahr (H–Y) stages I-II and 10 H–Y stage III, were studied. In healthy subjects, FBLoS decreased with aging and was lower in females. Mean (SD) FBLoS was 98.1 (48.9) Newtons (N) in healthy subjects, 70.5 (39.8) N in PD patients H–Y stages I-II, and 37.7 (18.9) N in PD patients H–Y stage III. Compared to healthy subjects and when adjusted for age and gender, PD patients H–Y stages I-II exhibited the following: (a) a reduced FBLoS; (b) larger CoP displacements and higher velocities for the same applied force; and (c) combined ankle and hip strategies elicited by less intense pull forces. All of these abnormalities were more pronounced in H–Y stage III PD patients compared to H–Y stages I-II PD patients. In conclusion, patients in the early stages of PD already exhibit a degree of postural instability due to inefficient postural adjustments, and they can more easily be destabilized by small perturbations than healthy subjects. This balance impairment becomes more pronounced in more advanced PD. In the pull test, pull force to step back should be a variable to consider when testing balance in clinical practice.

## 1. Introduction

Postural instability and gait disorders are among the main disabling and refractory-to-treatment symptoms in patients with advanced Parkinson's disease (PD). They constitute the main cause of falls, which are a frequent, recurrent, and transcendental phenomenon in PD associated with increased morbidity, mortality, deterioration of quality of life, and high health care financial costs [1–3]. Balance is defined as the ability to maintain the center of gravity (CoG) within the body's limits of stability (LoS), under both static and dynamic conditions [4]. Postural instability in PD is related to an impairment of postural reactive responses and a reduction of the functional LoS [5, 6].

The pull test or retropulsion test remains the main clinical test to assess balance in PD and other neurological disorders [7]. It is the only test that evaluates postural instability in the motor part of the Unified Parkinson's Disease Rating Scale (UPDRS) [8], and its pathological response indicates a change in the Hoehn & Yahr (H–Y) staging from II to III [9]. This progression implies clinical imbalance and, therefore, an increased risk of falls [10].

However, some studies have suggested that the accuracy of the pull test to predict falls is limited [11–14]. This may be partially because falling is the result of a complex interplay between balance, gait, cognition, and environmental factors, and the pull test only captures part of this [7, 15]. Another explanation lies in the interobserver variability of the pull test performance and interpretation. A recognized source of variability lies in the strength of the shoulder pull, in other words, there are as many different forces of the pull test as different examiners do the pulling [16].

In the original UPDRS, stepping backward in the pull test was classified as “retropulsion” [8]. However, the most recent MDS-UPDRS version specifies that the pull-force should sufficiently displace the CoG that the patient must take at least one step backward [17]. This practical consensus did not take into consideration the concept of the functional LoS. The LoS are defined as the maximum displacement of the body mass during a feet-in-place response to external perturbations without falling or stepping [18]. The LoS are reduced in PD patients, and this is considered a sign of postural instability [6]. Depending on the intensity and the characteristics of the pull force, stepping back, even a single step, could imply a reduction in the subject's backward LoS and, therefore, a sign of mild postural instability.

The pull test has not been examined systematically using reproducible mechanically produced forces. The purpose of our research was to perform a biomechanical analysis of the pull test in healthy subjects and PD patients. We hence assessed the force needed to reach the backward LoS, the displacements of the center of pressure, and the postural stabilizing strategies.

## 2. Materials and Methods

### 2.1. Participants

The study sample comprised 64 healthy subjects (38 females) who ranged broadly in terms of age, height, and weight in order to be able to adequately assess each of these physiological variables. The healthy subjects were mostly the PD patient's spouses or hospital staff who volunteered. Healthy subjects with any medical condition that could impair balance (e.g., blindness, vestibular pathology, history of stroke, peripheral neuropathy, cognitive impairment, significant spine pathology, or prior drug or alcohol abuse) were excluded. The inclusion criteria for a healthy participant were to be 18–90 y and do not suffer any of the excluded diseases. Six subjects were excluded (3 refused to participate and 3 presented an excluded pathology).

Thirty-two PD patients that fulfilled the UK Brain Bank Criteria [19], including H–Y stages I, II, and III, were studied. Patients were recruited from the outpatient clinic of the Movement Disorders Unit at Hospital Universitario Gregorio Marañón by a movement disorders specialized neurologist. PD patients with any other medical condition that could impair balance were similarly excluded. Patients treated with advanced therapies (neurosurgery, levodopa, or apomorphine pumps) and patients with moderate to severe dyskinesias that could alter the posturographic assessment were excluded. Eight PD patients were excluded (6 refused to participate, 1 presented an excluded pathology, and 1 presented H–Y stage IV).

Age, gender, height, weight, body mass index (BMI), medical and surgical history, current medication, and history of falls were assessed in all of the participants. An individual was considered as “faller” if he/she had suffered one or more falls in the previous 6 months. Fall was defined as an event which resulted in the patient unintentionally coming to the ground or other lower level not as a result of a major intrinsic event or overwhelming hazard [20]. In patients, the duration of the disease (from the onset of motor symptoms), the presence of motor fluctuations, the motor part of UPDRS [8], and the H–Y staging [9] were always recorded by the same clinician (JRPS) immediately prior to the test. All of the patients were on medication state when tested.

The study was approved by the local Ethics Committee of Hospital Universitario Gregorio Marañón. All participants signed an informed consent form.

### 2.2. Experimental Design

The participants were examined in the Posture and Gait Analysis Laboratory of our institution. To perform a mechanical pull test, we used a dedicated device with weights and pulleys (Rehab selection QM959-AL0; TechnoGym Inc.) connected to a harness surrounding the subject's shoulders that delivered, through a height adjustable cord set at midinterscapular line perpendicular to the body´s caudocranial axis, mechanically produced reproducible forces of 21.6 Newton (N), 36.3 N, 51.0 N, 65.7 N, 80.4 N, 95.1 N, 118.6 N, 142.1 N, 165.6 N, 189.1 N, 212.7 N, and 236.2 N.

The tests were performed over a posturographic force platform (AccuSway Plus; AMTI Inc.) to measure the center of pressure (CoP) displacements. Muscular activation patterns were studied by means of simultaneous surface electromyography (EMG) recordings from the following muscles: tibialis anterior, gastrocnemius lateralis, vastus lateralis, biceps femoris, rectus abdominis, and paravertebral muscles.

The participants were standing erect, eyes open, and barefooted with their feet 10–15 cm apart on the force platform. They had previously been informed about the nature of the procedure. Quick mechanically expected backward pulls were applied, with a progressive increase in the pull force (only a unique pull at each force level), until the subject took at least one step backward. Consecutive pulls were delivered at five-minute intervals to allow recovery from the previous set. Each participant was evaluated in a single session that lasted 60–90 minutes.

In every session, the following data were recorded:

1. The force to reach the backward limit of stability (FBLoS): the pull force needed to make the participant take at least one step backward.
2. The center of pressure (CoP) displacements: 19 posturographic variables regarding the reactive displacements of the CoP were obtained for each pull force applied. The posturographic variables are defined in Table 1.
3. The EMG recordings were triggered by the onset of every pull force that was delivered. The following variables were obtained:
  - Muscular activation latencies: time (msec) to the onset of the EMG activity recorded.
  - Postural stabilizing strategies: Ankle or hip strategies were identified according to the muscular activation pattern, as described by Horak and Nashner [21]. Ankle strategy was considered when anterior muscles (tibialis anterior, vastus lateralis, and rectus abdominis) activated before their corresponding posterior muscles at each level (gastrocnemius lateralis, biceps femoris, and paravertebral muscles, respectively) in a caudocranial direction. A combination of ankle and hip strategies was considered when there was simultaneous activation of posterior muscles (paravertebral muscles, biceps femoris, and gastrocnemius lateralis) and anterior muscles (tibialis anterior, vastus lateralis, and rectus abdominis).

### 2.3. Data Analysis

The descriptive results are expressed as means (SD) or medians [IQR] for continuous variables and as frequencies for the categorical variables. To determine the baseline differences between groups, chi-square test was used for the categorical variables, student's *t*-test for normal continuous variables, and Mann–Whitney *U* test for nonnormal continuous variables.

A hierarchical linear regression analysis was used to study the possible influence of the physiological variables age, gender, height, weight, and BMI (independent variables) on the FBLoS (dependent variable) in healthy subjects. BMI was included in a different model without height and weight to avoid multicollinearity. Multivariate linear regression models adjusted for the previous statistically significant physiological variables (covariates) were then performed to compare the FBLoS between groups (independent variable): healthy subjects vs. PD patients, healthy subjects vs. PD H–Y stages I-II, PD stages I-II vs. PD H–Y stage III, and PD nonfallers vs. PD fallers. The FBLoS variable was log transformed (dependent variable) to improve the normal distribution of the variable and the statistical models.

Spearman's rho test was used to correlate the different forces tested for each subject with the reactive displacements of the CoP. To analyze the displacements of CoP, a multivariate analysis was performed for each posturographic variable (dependent variable), adjusted for force, age, and gender (covariates), between groups (independent variable): healthy subjects vs. PD patients, healthy subjects vs. PD H–Y stages I-II, and PD stages I-II vs. PD H–Y stage III.

The muscular activation latencies (dependent variables) were analyzed with multivariate models controlled for force, age, and gender (covariates) between groups (independent variable): healthy subjects vs. PD patients, healthy subjects vs. PD H–Y stages I-II, and PD stages I-II vs. PD H–Y stage III.

The chi-square test was used to compare the stabilizing strategies between groups.

A *P*^–^value <0.05 was considered statistically significant.

## 3. Results

The clinical and demographic details of the healthy subjects, the PD patients and their subgroups, descriptive data from the study variables, the FBLoS, posturographic variables, muscular latencies, and stabilizing strategies are summarized in Table 2.

The results of the statistical analysis between the different groups in terms of the FBLoS, posturographic variables, and muscular latencies are presented in Table 3.

### 3.1. Force to Reach the Limit of Stability

In healthy subjects, the FBLoS decreased with increasing age (*P*=0.007). There was a mean reduction of 9.9 N in the FBLoS for every decade of life. The FBLoS was also lower in females (*P*=0.008), and it was not affected by the weight (*P*=0.664), height (*P*=0.488), or BMI (*P*=0.689) of the subjects.

**Figure 1:**
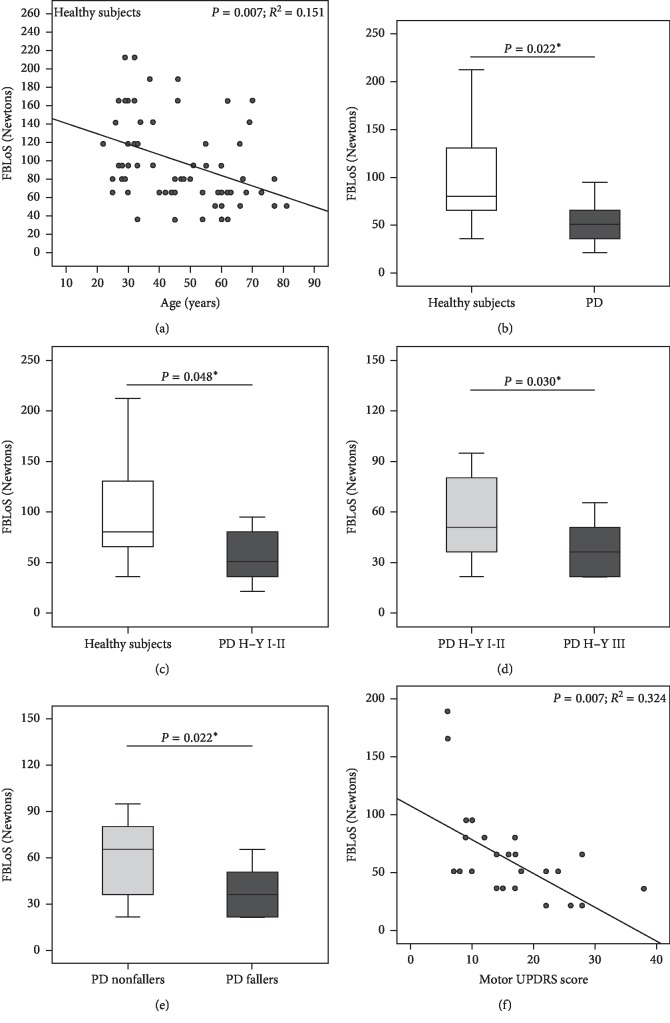
Force to reach the backward limit of stability (FBLoS) in the analyzed groups. PD: Parkinson's disease patients. H–Y: Hoehn–Yahr. ^*∗*^Adjusted for age and gender. (a) Association between FBLoS and age in healthy subjects. (b) Comparison of FBLoS between healthy subjects and PD patients. (c) Comparison of FBLoS between healthy subjects and PD H–Y stages I-II. (d) Comparison of FBLoS between PD H–Y stages I-II and PD H–Y stage III. (e) Comparison of FBLoS between PD nonfallers and PD fallers. (f) Association between FBLoS and motor UPDRS score in PD patients.

The age- and gender-adjusted FBLoS was lower in the PD patients than in the healthy subjects (*P*=0.022). For example, according to this multivariate model, a seventy-year-old PD male patient would reach his backward limit of stability with a pull force 20.5 N lower than an age- and gender-matched healthy subject. The FBLoS was significantly lower in the early stages of PD (H–Y I and II) compared with healthy subjects (*P*=0.048). In PD patients, the FBLoS was reduced in H–Y stage III compared to H–Y stages I-II (*P*=0.030). In addition, there was a linear decrease in the FBLoS with higher motor UPDRS scores (*P*=0.007), and in the PD fallers, the FBLoS was significantly lower than in the PD nonfallers (*P*=0.022). The mean difference in the FBLoS between the fallers and the nonfallers was 31.1 (13.9) N. Graphs illustrating these comparisons are shown in Figure 1.

### 3.2. Center of Pressure Displacements

**Figure 2:**
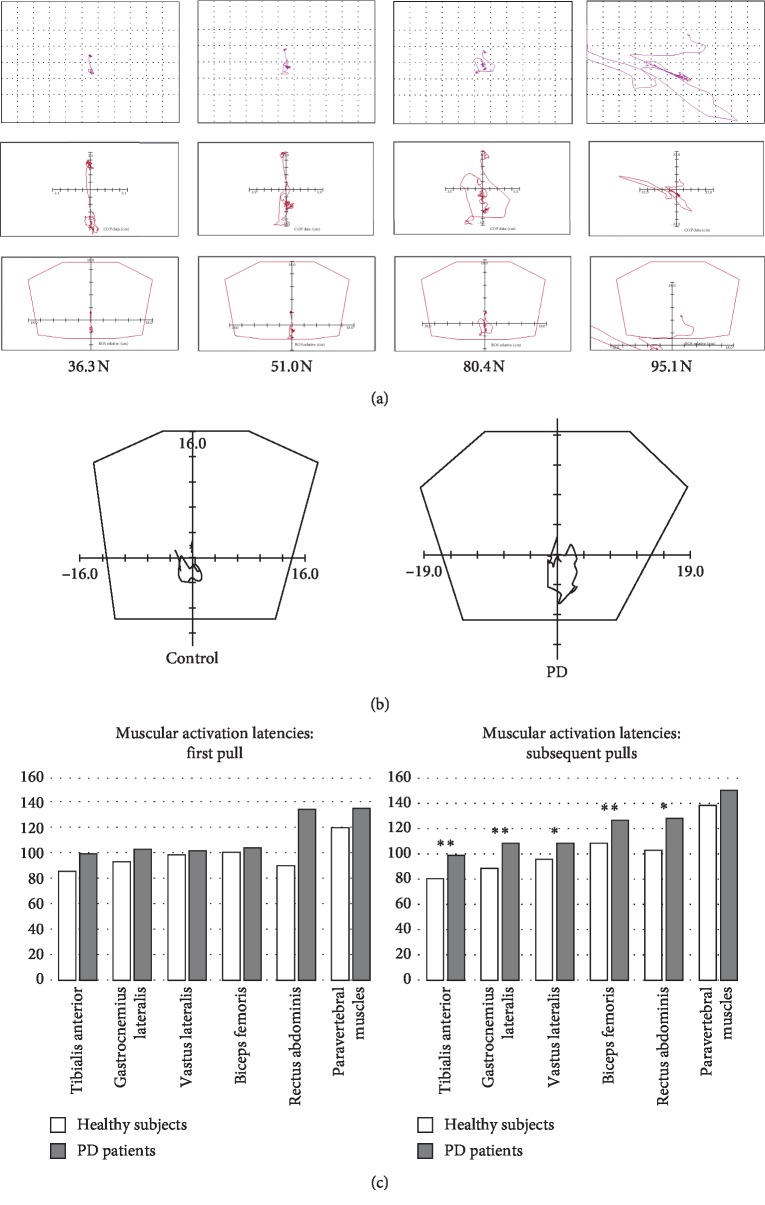
(a) Posturography example of a 55 y old healthy female: The greater pull force delivered the greater displacements of the center of pressure (CoP) provoked. (b) Example of the CoP displacements for a force of 51.0 N in a healthy subject and an age- and gender-matched PD patient, which shows a larger area of CoP displacement than the healthy subject. (c) Mean muscular latencies in healthy subjects and PD patients for the first pull and subsequent pulls. ^*∗*^*P* < 0.05^*∗∗*^*P* < 0.01.

There was a positive correlation between the force delivered and the ensuing displacements of CoP. An *r* > 0.3 (Spearman's rho; *P* < 0.001) was found for 17 of the 19 studied posturographic variables (Table 1), and *r* ≥ 0.5 (*P* < 0.001) for 8 of the variables. A graphic example of this correlation is shown in Figure 2(a).

After adjusting for force, age, and gender, the PD patients exhibited larger displacements of CoP than the healthy subjects in the average lateral axis (*X* avg) (*P*=0.009) and the maximum forward and backward displacements (*Y* ant and *Y* post) (*P*=0.043 and *P* < 0.001, respectively), increased CoP backward velocities (*Vy* post) (*P*=0.001) and the average velocity (*V* avg) (*P*=0.002), greater total distance covered by the CoP (path length) (*P*=0.002), increased CoP displacement areas (rectangular area and area95) (*P*=0.044 and *P*=0.049, respectively), and the length of the major axis of the 95^th^ percentile ellipse (major95) (*P*=0.035). A graphic example is shown in Figure 2(b). Similar differences were observed in the early stages of PD (H–Y I and II) compared with healthy subjects. In addition, PD H–Y stage III patients exhibited larger maximum mediolateral displacements (*X* right and *X* left) (*P*=0.008 and *P*=0.013, respectively) and larger CoP displacement areas (rectangular area and area95) (*P*=0.010 and *P*=0.021, respectively) than PD H–Y stage I-II patients.

### 3.3. Muscular Latencies and Stabilizing Strategies

The muscular activation latencies elicited by the first pull were larger in the PD patients than in the healthy subjects for the tibialis anterior (*P*=0.013). For the subsequent pulls, the PD patients also exhibited larger latencies for the tibialis anterior, gastrocnemius lateralis, vastus lateralis, biceps femoris, and rectus abdominis (*P* < 0.05). In the multivariate analysis, after adjusting for force, age, and gender, there were no significant differences in the latencies between the PD patients and the healthy subjects for the first pull. However, for the subsequent pulls, the latency values were still significantly larger in the PD patients for the tibialis anterior, gastrocnemius lateralis, vastus lateralis, biceps femoris, and rectus abdominis muscles (*P* < 0.05). A graph of the latencies is shown in Figure 2(c). Delayed muscular activation with the second and subsequent pulls was already noted in PD patients with H–Y stages I-II compared with healthy subjects in all muscles (*P* < 0.05) except for the paravertebral muscles. There was not a significant difference in the muscular latencies between the PD patients with H–Y stages I-II and H–Y III for either the first pull or for the subsequent pulls (*P* > 0.05).

In the group of healthy subjects, the EMG assessment of the pattern of muscle activation revealed that an isolated ankle postural strategy occurred in 80.4% of the tests performed (254/316) and the combination of an ankle and hip strategy occurred in 19.6% of the tests (62/316). In the PD group, an isolated ankle strategy was recorded in 60.4% of the tests (61/101) and a combined hip and ankle strategy in 39.6% of the tests (40/101). The differences between the healthy subjects and the PD group were significant (*P* < 0.001). The PD patients with H^–^Y stages I-II also exhibited a higher proportion of combined hip and ankle strategies than the healthy subjects (35.8% vs. 19.6%; *P*=0.002). The lowest pull force (21.6 N) elicited an ankle strategy in the healthy subjects and the PD patients. In the healthy subjects, the mean force to induce a combined ankle and hip strategy was 78.1 (42.9) N, whereas in PD patients, it was 54.1 (33.2) N (*P* < 0.001) [59.2 (35.0) N in the early PD H–Y stages I-II and 35.4 (15.5) N in PD stage H–Y III (*P*=0.001)]. After adjusting for age and gender in a multivariate analysis, the differences observed in the force to induce a combined ankle and hip strategy were significant between the healthy subjects and the PD H–Y I-II patients (*P*=0.045) and also between the PD H–Y I-II and the PD H–Y III patients (*P*=0.012).

## 4. Discussion

To gain more insight regarding the reactive postural responses elicited by reproducible pull forces, we designed a multimodal study of the pull test paradigm that included the pull forces needed to reach the backward limit of stability, the displacements of the center of pressure recorded on a force platform, and the latencies and patterns of activation of the stabilizing muscles. To our knowledge, this is the first systematic study of the pull test in healthy subjects and PD patients with such approach, using reproducible mechanically produced forces.

### 4.1. Force to Reach the Backward Limit of Stability

In healthy subjects, the FBLoS diminished progressively with age (9.9 N per decade). Reduction in the FBLoS in the elderly is a plausible biological finding. It is well known that the biomechanical and neurophysiological systems involved in balance deteriorate with age, both in terms of the afferent components (visual, vestibular, and proprioception) and the efferent components (musculoskeletal system and viscoelastic properties of ligaments, tendons, and junctions) [22–24].

In addition, we found that females had lower FBLoS values. This finding may be related to gender differences in muscle strength [25]. Weight and BMI did not have a significant independent influence on the FBLoS.

The FBLoS in PD patients, adjusted for age and gender, was lower than in healthy subjects. In patients with PD, the FBLoS diminished with disease progression (Hoehn–Yahr staging and motor UPDRS) and was 31.1 N lower in fallers.

These results imply that it is more likely that parkinsonian patients may be destabilized by small external perturbations than healthy subjects, particularly patients with more advanced disease and those who have experienced falls.

An interesting finding from this study is that the FBLoS was already reduced in patients in Hoehn–Yahr stages I and II. This suggests that even PD patients in the early stages of the disease have impaired postural adjustments to maintain the base of support when exposed to potentially destabilizing external forces. Thus, a mild balance impairment could already occur in the first years of the disease, and this may play a role in falls reported by patients before they have reached Hoehn–Yahr stage III [10].

Few studies to date have tried to assess the FBLoS in PD in terms such as “pulling a subject off-balance” [26] or the “stepping threshold” [27]. In these studies, no significant differences were found between patients and controls. However, methodological differences—e.g., the pull rope being attached at the lumbar level, as it is closer to the human body's center of gravity [26], pulls for which the direction and the force vary, which avoids the learning component [27], exclusion of patients with a history of falls, and a small number of subjects studied—could explain the difference in results.

### 4.2. Center of Pressure Displacements

For the same force applied in the pull test, PD patients had larger CoP displacements and a higher average and backward velocities than healthy subjects, when corrected for age and gender. This abnormal postural sway induced by pull forces suggests that parkinsonian patients need more postural adjustments to relocate the body's center of gravity within their limits of stability than normal subjects, and this could be part of the mechanisms underlying postural instability in PD. Moreover, large displacements of the CoP in the mediolateral direction, which were also found in our study in parkinsonian patients, have been related to falls in PD [28, 29].

Our results are in keeping with those of previous studies that found increased postural sway in static and dynamic conditions in PD patients [28, 30–32]. However, to our knowledge, this is the first study to demonstrate an increase in CoP displacements using reproducible forces in a biomechanical pull test paradigm.

It is noteworthy that larger CoP displacements and higher velocities were already detected in patients with Hoehn–Yahr stages I and II compared to normal subjects, and a more pronounced postural sway in patients with Hoehn–Yahr stage III. These results, along with observations from other groups using trunk accelerometry [33] confirm that PD patients experience an abnormal postural sway before clinical balance impairment becomes manifest.

### 4.3. Muscular Latencies and Stabilizing Strategies

PD patients and control subjects exhibited an ankle strategy with low pull forces. Latencies for the first activated muscle, the tibialis anterior, elicited by the first pull force were similar in both groups.

Previous studies [34] did not find differences in muscular latencies in response to surface translations between PD patients and healthy controls.

However, in this study, with subsequent pulls, control subjects had shorter latencies, whereas the latencies for muscle activation in PD patients remained unchanged. This shortening of the latencies for activation of the stabilizing muscles after repeated pull tests could be related to a possible adaptive response to expected perturbations in normal subjects, while PD patients did not exhibit signs of this adaptation. This observation may be in line with the concept of motor learning impairment in PD patients [35].

In control subjects, a combination of ankle and hip strategies was only observed when intense pull forces were applied. However, in PD patients, combined hip and ankle strategies were more frequent and elicited by less intense forces, even in the early stages of the disease. This precocious recruitment of both postural strategies in patients with PD could be a compensatory mechanism of an already impaired balance system.

### 4.4. Clinical Implications and Limitations

Our results have clinical implications. When assessing balance in PD patients with the pull test, a single-step backward as a response to a low-intensity pull force may be an early indication of impairment of postural responses. Thus, in the pull test, pull force to step back should be a variable to consider when testing balance in clinical practice. On the contrary, the physical therapy approaches intended to improve balance that are commonly offered to PD patients with overt instability and falls could be considered for patients with mild to moderate stages of the disease.

A possible limitation of this study could be the sample size of the Hoehn–Yahr stage III PD patients. However, the results for this group were robust and consistent despite the relatively small number of cases.

In summary, taking together, we found that patients in the early stages of PD, i.e., Hoehn–Yahr stages I and II, already exhibit signs of postural instability. These patients, when exposed to pull forces, experienced an abnormal postural sway. To try to compensate for the destabilizing effect induced by the external perturbation, in addition to the usual postural ankle strategy, PD patients also precociously recruited the postural hip strategy. Despite the combined postural strategies, PD patients had lower FBLoS, which meant that they could be destabilized by smaller pull forces than healthy subjects. At this point, they had to activate the rescue strategy of stepping back to modify the base of support and to avoid falling.

## 5. Conclusion

Postural instability in PD could be considered to be a continuum, ranging from a mild impairment of balance already present in Hoehn–Yahr stages I and II to severe postural instability in more advanced stages of the disease. When testing balance in PD patients in clinical practice with the pull test, pull force to step back should be taken into consideration since a single step backwards as a response to a low-intensity pull may indicate early impairment of postural responses.

## Figures and Tables

**Table 1:** Posturographic variables.

Posturographic variables	Definition
*General statistics (cm):*	
*X* avg	Average displacement of the CoP in the lateral (*X*) axis of the platform
*X* right	Maximum displacement of the CoP in the right direction
*X* left	Maximum displacement of the CoP in the left direction
*X* SD	Standard deviation of the mean displacement of the CoP in the lateral (*X*) axis
*Y* avg	Average displacement of the CoP in the anterior-posterior (*Y*) axis of the platform
*Y* ant	Maximum displacement of the CoP in the anterior direction
*Y* post	Maximum displacement of the CoP in the posterior direction
*Y* SD	Standard deviation of the mean displacement of the CoP in the anterior-posterior (*Y*) axis

*Velocity measures (cm/s)*	
*Vx* right	Maximum velocity of the CoP in the right direction
*Vx* left	Maximum velocity of the CoP in the left direction
*Vy* ant	Maximum velocity of the CoP in the anterior direction
*Vy* post	Maximum velocity of the CoP in the posterior direction
*V* avg	Average velocity of the CoP

*Sway areas (cm* ^*2*^)	
Rectangular area	Rectangular area that encompasses 100% of the data
Circular area	Circular area that encompasses 100% of the data
Area95	95^th^ percentile of an ellipse fitted to the overall CoP trace
Major95 (cm)	Length of the major axis of the 95^th^ percentile ellipse
Minor95 (cm)	Length of the minor axis of the 95^th^ percentile ellipse
Path length (cm)	Total distance covered by the CoP

CoP: center of pressure; cm: centimeters; s: seconds.

**Table 2:** Clinical and demographic details of healthy subjects and PD patients and their subgroups, descriptive data from the study variables, force to reach the backward limit of stability, posturographic variables, and muscular latencies.

Variable	HS	PD	HS vs. PD *P* value	PD H–Y I-II	HS vs. PD I-II *P* value	PD H–Y III	PD I-II vs. PD III *P* value
Number of subjects	64	32		22		10	
Age (y)	45 [22–81]	72 [45–86]	<0.001	69.5 [45–82]	<0.001	73 [69–86]	0.023
Women	38 (59%)	15 (47%)	0.246	9 (41%)	0.189	6 (60%)	0.316
Weight (kg)	68.4 (12.7)	71.0 (12.8)	0.240	72.0 (13.2)	0.183	68.9 (12.1)	0.529
Height (cm)	167.5 (11.3)	166.1 (8.7)	0.926	168.2 (6.9)	0.455	161.4 (10.8)	0.097
BMI (kg/m²)	24.6 (4.0)	25.7 (3.7)	0.108	25.3 (3.1)	0.138	26.6 (4.8)	1.0
Motor UPDRS	No	16.4 (8.0)	—	12.4 (3.9)	—	25.2 (7.7)	<0.001
Disease duration (y)	No	6.4 (4.2)	—	5.3 (4.0)	—	8.7 (4.0)	0.017
Motor fluctuations	No	9 (28.1%)	—	4 (18.2%)	—	5 (50%)	0.064
Fallers	No	9 (28.1%)	—	2 (9.1%)	—	7 (70%)	<0.001
FBLoS (N)	98.1 (48.9)	60.2 (37.7)	<0.001	70.5 (39.8)	0.004	37.7 (18.9)	0.023

*Posturographic variables*							
*X* avg (cm)	0.7 [0.3–1.3]	0.9 [0.5–2.0]	0.001	0.9 [0.5–1.8]	0.020	1.9 [1.2–2.4]	0.029
*X* right (cm)	1.3 [0.9–1.8]	1.4 [1.0–2.2]	0.048	1.4 [1.0–2.1]	0.048	1.3 [0.9–3.7]	0.924
*X* left (cm)	1.2 [0.9–1.8]	1.3 [1.0–1.9]	0.261	1.3 [1.0–1.9]	0.307	1.3 [0.9–3.1]	0.825
*X* SD (cm)	0.5 [0.4–0.8]	0.6 [0.5–0.8]	0.045	0.6 [0.5–0.8]	0.073	0.6 [0.4–1.2]	0.674
*Y* avg (cm)	6.9 [5.4–9.0]	3.8 [2.2–5.5]	<0.001	3.8 [3.0–5.4]	<0.001	1.8 [0.6–7.6]	0.068
*Y* ant (cm)	3.2 [2.5–4.0]	3.2 [2.1–4.7]	0.692	3.3 [2.2–4.8]	0.411	3.0 [1.6–3.5]	0.216
*Y* post (cm)	3.3 [2.7–4.1]	3.9 [3.1–5.2]	<0.001	3.9 [3.1–5.3]	<0.001	4.0 [2.8–5.3]	0.894
*Y* SD (cm)	1.9 [1.5–2.4]	2.2 [1.5–2.9]	0.015	2.3 [1.5–3.0]	0.016	2.1 [1.6–2.4]	0.493
*Vx* right (cm/s)	13.3 [8.1–21.5]	14.3 [8.5–23.4]	0.677	14.3 [8.4–23.3]	0.767	12.9 [8.3–56.2]	0.735
*Vx* left (cm/s)	20.1 [13.1–33.0]	21.6 [13.5–33.7]	0.684	21.6 [13.6–34.0]	0.639	21.6 [11.5–29.7]	0.691
*Vy* ant (cm/s)	13.6 [8.3–22.2]	15.1 [9.2–23.3]	0.289	14.9 [9.3–22.4]	0.464	19.7 [7.8–39.4]	0.295
*Vy* post (cm/s)	40.3 [28.7–56.1]	40.7 [25.0–61.1]	0.775	43.2 [26.0–61.3]	0.445	29.4 [23.5–48.2]	0.238
*V* avg (cm/s)	3.2 [2.5–3.9]	3.2 [2.7–4.4]	0.137	3.2 [2.7–4.4]	0.147	3.3 [2.8–4.2]	1.000
Rectangular area (cm²)	17.6 [10.9–26.1]	20.4 [12.6–35.3]	0.018	20.6 [13.5–35.0]	0.015	15.2 [9.7–53.8]	0.791
Circular area (cm²)	8.3 [5.5–12.4]	8.4 [5.8–14.9]	0.179	8.7 [5.8–14.8]	0.218	7.7 [5.3–28.3]	0.871
Area95 (cm²)	17.3 [10.7–27.6]	20.9 [13.1–34.8]	0.034	20.9 [13.4–33.9]	0.034	17.5 [11.3–49.0]	0.918
Major95 (cm)	4.7 [3.9–5.9]	5.6 [3.9–7.2]	0.014	5.7 [3.8–7.3]	0.015	5.2 [4.0–6.1]	0.470
Minor95 (cm)	1.2 [0.9–1.6]	1.3 [0.9–1.7]	0.196	1.3 [0.9–1.7]	0.221	1.1 [0.8–2.8]	0.930
Path length (cm)	31.6 [24.6–39.3]	32.2 [26.8–44.0]	0.133	32.1 [26.8–44.2]	0.143	32.5 [27.5–42.1]	1.000

*Muscular latencies (msec)*							
*First pull:*							
Tibialis anterior	85.9 (19.0)	99.8 (23.6)	0.013	100.5 (27.4)	0.026	98.2 (13.2)	0.819
Gastrocnemius lateralis	93.9 (24.3)	103.7 (28.4)	0.206	105.4 (33.5)	0.210	99.6 (8.8)	0.513
Vastus lateralis	98.9 (16.9)	102.0 (23.8)	0.652	103.9 (27.3)	0.549	97.3 (11.8)	0.454
Biceps femoris	101.1 (15.5)	105.3 (23.1)	0.609	109.0 (25.3)	0.390	95.0 (12.9)	0.189
Rectus abdominis	90.7 (31.7)	135.5 (36.3)	0.061	132.3 (38.2)	0.055	140.2 (10.1)	0.412
Paravertebral muscles	120.6 (43.8)	136.1 (23.8)	0.499	125.8 (14.1)	0.814	150.0 (30.0)	0.207

*Subsequent pulls:*							
Tibialis anterior	80.3 (19.1)	98.3 (24.6)	<0.001	98.7 (24.9)	<0.001	95.5 (24.0)	0.701
Gastrocnemius lateralis	88.8 (22.5)	107.9 (26.7)	<0.001	107.8 (26.9)	<0.001	108.9 (27.0)	0.910
Vastus lateralis	95.9 (22.5)	109.3 (31.5)	0.004	110.9 (32.3)	0.002	95.0 (20.0)	0.208
Biceps femoris	108.7 (26.1)	126.6 (31.9)	<0.001	127.8 (33.1)	<0.001	114.0 (6.2)	0.014
Rectus abdominis	102.8 (28.3)	128.2 (30.7)	<0.001	127.3 (30.5)	<0.001	165.0 (32.1)	0.230
Paravertebral muscles	138.3 (45.1)	149.9 (55.1)	0.196	150.3 (55.9)	0.190	143.3 (49.2)	0.834

Data are presented as means (SD) and medians [IQR] for continuous variables. Age is presented with median [minimum-maximum]. Qualitative variables are expressed as frequencies. *P* values were obtained using the Mann–Whitney *U* test for nonnormal continuous variables, the Student's *t*-test for normal continuous variables, and the chi-square test for categorical variables. HS: healthy subjects; PD: Parkinson's disease patients; y: years; kg: kilograms; cm: centimeters; msec: milliseconds; UPDRS: Unified Parkinson's Disease Rating Scale; FBLoS: force to reach the backward limit of stability; N: Newton.

**Table 3:** Multivariate analyses. Comparisons of force to reach the backward limit of stability, posturographic variables, and muscular latencies between the different groups.

	HS vs. PD			HS vs. PD H–Y I-II			PD H–Y I-II vs. PD H–Y III		
	*R*²	*β*	*P* value	*R*²	*β*	*P* value	*R*²	*β*	*P* value
FBLoS^*∗*^	0.447	−0.229	**0.022**	0.395	−0.210	**0.048**	0.437	−0.357	**0.030**

*Posturographic variables* ^*∗∗*^									
*X* avg	0.046	0.159	**0.009**	0.032	0.128	**0.035**	0.251	−0.001	0.993
*X* right	0.027	0.085	0.167	0.029	0.056	0.354	0.198	0.303	**0.008**
*X* left	0.025	0.037	0.545	0.030	−0.002	0.977	0.099	0.299	**0.013**
*X* SD	0.027	0.080	0.192	0.031	0.048	0.428	0.091	0.258	**0.032**
*Y* avg	0.229	−0.298	**<0.001**	0.207	−0.282	**<0.001**	0.085	−0.142	0.234
*Y* ant	0.073	0.122	**0.043**	0.075	0.136	**0.021**	0.233	0.015	0.889
*Y* post	0.084	0.227	**<0.001**	0.079	0.225	**<0.001**	0.122	0.154	0.189
*Y* SD	0.075	0.130	**0.030**	0.077	0.138	**0.019**	0.258	0.087	0.416
*Vx* right	0.021	0.016	0.801	0.032	−0.023	0.708	0.227	0.058	0.531
*Vx* left	0.040	0.017	0.780	0.039	0.018	0.764	0.214	0.045	0.685
*Vy* ant	0.018	0.023	0.712	0.022	−0.003	0.957	0.211	0.378	**0.001**
*Vy* post	0.199	0.188	**0.001**	0.223	0.183	**0.001**	0.422	0.141	0.138
*V* avg	0.056	0.190	**0.002**	0.058	0.180	**0.003**	0.239	0.123	0.261
Rectangular area	0.032	0.124	**0.044**	0.031	0.096	0.114	0.159	0.303	**0.010**
Circular area	0.043	0.102	0.095	0.043	0.084	0.161	0.174	0.224	0.050
Area95	0.041	0.120	**0.049**	0.044	0.092	0.124	0.113	0.276	**0.021**
Major95	0.070	0.127	**0.035**	0.073	0.135	**0.023**	0.253	0.086	0.426
Minor95	0.046	0.094	0.125	0.057	0.056	0.350	0.105	0.288	**0.017**
Path length	0.055	0.189	**0.002**	0.057	0.180	**0.003**	0.239	0.123	0.261

*Muscular latencies* ^*∗∗*^									
First pull									
Tibialis anterior	0.119	0.262	0.073	0.108	0.263	0.083	0.174	−0.093	0.643
Gastrocnemius lateralis	0.043	0.156	0.361	0.048	0.172	0.331	0.153	−0.088	0.703
Vastus lateralis	0.030	0.013	0.984	0.033	0.050	0.811	0.181	−0.203	0.434
Biceps femoris	0.035	0.123	0.656	0.057	0.140	0.624	0.225	−0.474	0.143
Rectus abdominis	0.677	0.379	0.240	0.690	0.399	0.202	0.608	0.503	0.345
Paravertebral muscles	0.607	0.449	0.180	0.582	0.394	0.317	0.590	0.631	0.238

*Subsequent pulls*									
Tibialis anterior	0.204	0.267	**<0.001**	0.210	0.276	**<0.001**	0.364	−0.088	0.421
Gastrocnemius lateralis	0.170	0.254	**0.001**	0.165	0.254	**0.001**	0.357	−0.023	0.835
Vastus lateralis	0.085	0.228	**0.011**	0.096	0.241	**0.007**	0.266	−0.102	0.399
Biceps femoris	0.099	0.252	**0.005**	0.107	0.257	**0.004**	0.259	−0.092	0.470
Rectus abdominis	0.268	0.188	**0.042**	0.255	0.184	**0.049**	0.251	0.161	0.291
Paravertebral muscles	0.022	0.081	0.441	0.024	0.087	0.409	0.075	−0.029	0.853

This table summarizes the regression models used in the study. The left column shows all the dependent variables, and the upper row shows the different groups used as independent variables in each model. ^*∗*^Controlled for age and gender; ^*∗∗*^controlled for force, age, and gender; *R*^2^: value of the complete regression model; *β*: *β*-coefficient; FBLoS: force to reach the backward limit of stability; HS: healthy subjects; PD: Parkinson's disease patients; H–Y: Hoenh & Yahr. *P* values correspond to listed variables and groups. Definition of posturographic variables is indicated in Table 1.

## Data Availability

The data used to support the findings of this study are included within the article. Additional data of this study have been deposited at Universidad Complutense de Madrid in the PhD studies repository: https://eprints.ucm.es/50108/1/T40642.pdf
